# Autoantibody Profiling for Lung Cancer Screening Longitudinal Retrospective Analysis of CT Screening Cohorts

**DOI:** 10.1371/journal.pone.0087947

**Published:** 2014-02-03

**Authors:** Kourtney Trudgen, Nada H. Khattar, Eric Bensadoun, Susanne Arnold, Arnold J. Stromberg, Edward A. Hirschowitz

**Affiliations:** 1 Division of Pulmonary and Critical Care Medicine, University of Kentucky, Lexington, Kentucky, United States of America; 2 Division of Medical Oncology, University of Kentucky, Lexington, Kentucky, United States of America; 3 Department of Statistics, University of Kentucky, Lexington, Kentucky, United States of America; 4 Lexington Veteran’s Administration Medical Center, Lexington, Kentucky, United States of America; San Raffaele Scientific Institute, Italy

## Abstract

Recommendations for lung cancer screening present a tangible opportunity to integrate predictive blood-based assays with radiographic imaging. This study compares performance of autoantibody markers from prior discovery in sample cohorts from two CT screening trials. One-hundred eighty non-cancer and 6 prevalence and 44 incidence cancer cases detected in the Mayo Lung Screening Trial were tested using a panel of six autoantibody markers to define a normal range and assign cutoff values for class prediction. A cutoff for minimal specificity and best achievable sensitivity were applied to 256 samples drawn annually for three years from 95 participants in the Kentucky Lung Screening Trial. Data revealed a discrepancy in quantile distribution between the two apparently comparable sample sets, which skewed the assay’s dynamic range towards specificity. This cutoff offered 43% specificity (102/237) in the control group and accurately classified 11/19 lung cancer samples (58%), which included 4/5 cancers at time of radiographic detection (80%), and 50% of occult cancers up to five years prior to diagnosis. An apparent ceiling in assay sensitivity is likely to limit the utility of this assay in a conventional screening paradigm. Pre-analytical bias introduced by sample age, handling or storage remains a practical concern during development, validation and implementation of autoantibody assays. This report does not draw conclusions about other logical applications for autoantibody profiling in lung cancer diagnosis and management, nor its potential when combined with other biomarkers that might improve overall predictive accuracy.

## Introduction

Results from the 10-year National Lung Screening Trial (NLST) show low dose CT screening confers a survival benefit in the at-risk population [1]. Although radiographic imaging is the de-facto screening modality, circulating biomarkers have potential to enhance early detection initiatives and further improve outcomes [2]–[6]. Our group and others have been developing autoantibody assays that could complement CT scanning in lung cancer diagnosis and management [4]–[9]. It is now well established that cancer patients produce autoantibodies to tumor proteins that are mutated, misfolded, ectopically presented, over-expressed, aberrantly degraded or anomalously glycosylated [4]–[13]. Assays comprised of panels of robust and complementary markers selected from an extensive repertoire of tumor-associated antibodies are designed to compensate for tumor heterogeneity. Biological amplification of low frequency cellular aberrancy makes autoantibodies logical biomarkers for early detection and a prevailing strategy for detecting occult malignancy [2]–[15]. Six markers from prior discovery were analyzed in a comparative study using samples from two independent CT screening studies. Integrity and relative comparability of two screening sample cohorts from, each with a high percentage of cancer samples drawn prior to radiographic detection, offered a unique opportunity to test principles, precepts, and dominant objectives of investigation to date [7]–[9], [16]–[18].

A panel of six autoantibody markers were used to assay samples from the Mayo Clinic CT screening trial, to gather normal distribution values, and generate a cutoff value that might be used to improve efficiency of lung cancer screening. Established cutoff values were applied to 285 samples from 95 participants of a regional CT screening study in the 5^th^ district of Kentucky (Appalachia). The primary objective of the study was to determine the ability of an autoantibody profile to detect lung cancers at the time of or before CT scan. The uniformity of sample collection and study entry criteria was an important standard for analysis within and between the two screening sample cohorts. Class prediction in sample sets comprised predominantly of occult lung cancers (prior to radiographic detection) is a unique aspect of this analysis. Accurate classification of stage I screening detected cancers was a secondary metric.

## Materials and Methods

### Ethics Statement

Samples were collected under protocols approved by accredited Institutional Review Boards (Mayo Clinic IRB and University of Kentucky IRB). All subjects provided written informed consent prior to any research procedures. This research was approved by respective IRBs and was conducted according to Institutional Review Board regulations and oversight.

### Mayo cohort

The Mayo Lung Screening Trial performed five annual CTs on 1520 subjects with a minimum 20 pack-year smoking history, age 50–75, and no other malignancy within five years of study entry [16], [17]. Cancer rates were 2.6% at 3 years rising to 4% at 5 years of screening. A single blood sample was drawn at study entry. The sample cohort was comprised of 180 non-cancer controls, six stage I prevalence lung cancers, and 44 lung cancers diagnosed 12 to 60 months from blood draw [16], [17].

### Kentucky cohort

The Marty Driesler Lung Screening Project was a community-based CT screening study that accrued 254 at risk subjects from Eastern Kentucky between 2005 and 2008 [18]. Eligibility criteria included age 55 to 75 years, 30 pack-years history of smoking, and no other malignancy within five years of study entry. Cancer rate was 2.6%. All subjects provided written informed consent prior to any research procedures.

Since analysis of all available samples was cost prohibitive, a sample set of two hundred fifty six samples from ninety-five participants was constructed by an independent investigator and analyzed in a blinded fashion. The test cohort of nineteen lung cancer samples included five stage I screening detected lung cancers (three prevalence, two incidence), and four lung cancers diagnosed clinically one to five years after the last serial screening CT and corresponding blood sample. One case of head and neck cancer was diagnosed during the screening period, and six other non-thoracic malignancies were diagnosed up to five years from the last lung cancer screening CT. All cancer cases are summarized in Table 1. One or more non-malignant pulmonary nodules were noted in 56% of the study cohort. Dominant non-malignant radiographic findings included emphysema, mediastinal adenopathy and granulomatous disease.

**Table 1:** Characteristics of cancers associated with the KY screening cohort.

Cancer.	Histology	Stage	Sample-year (screening)	Lead time to diagnosis (months)	Prediction (<640fu)
**Screening-detected Lung Cancers**					
Prevalence	AdenoCa	IA	1	0	-
Prevalence	AdenoCa	IA	1	0	+
Prevalence	AdenoCa	IA	1	0	+
Incidence	Squamous	IA	1, 2, 3	24/12/0	+/+/+
Incidence	Squamous	IIB	1, 2, 3	29/13/0	+/+/+
**Clinically-detected Lung Cancers (post-screening)**					
Incidental	Squamous	IB	1, 2, 3	41/28/14	+/+/+
Incidental	AdenoCa	IB	1, 2, 3	45/32/20	–/–/–
Incidental	Squamous	IB	1, 2, 3	57/44/31	–/–/–
Incidental	NSCLC	IIIB	1	28	-
**Other Cancers** *					
B-Cell Lymphoma(MALT)	Extranodal Marginal Zone Lymphoma	IIEA	1	59	+
Colon	AdenoCa	IV	1, 2, 3	50/38/25	+/+/+
Head and Neck	Squamous	I	1, 2, 3	9/+3/+15	–/+/–
Head and Neck	Carcinoma (NOS)	IIB	1	31	–
Histocytic Sarcoma (tonsil)	Follicular Dendritic Cell Sarcoma (FDCS)	unknown	1, 2	37/25	+/+
Breast	AdenoCa	0 (CIS)	1, 2, 3	53/39/27	+/+/+
Bladder	Papillary	0 (CIS)	1, 2, 3	27/15/2	+/+/+

* Exclusion criteria included: (1) Current or prior personal history of lung cancer (2) Prior malignancy except adequately treated non-melanomatous skin cancer or in-situ cervical cancer.

The table includes class prediction and temporal relationship of sample draw to cancer diagnosis. Binomial prediction is based on additive measures from the six-marker panel. Up to three individual sample measures from each subject are designated either positive (+) or negative (–) based on levels relative to a predetermined cutoff value of 640 FU (fluorescent units). Assay results at time-of-diagnosis (radiographic detection) of five screening detected lung cancers (three prevalence and two incidence cancers) are designated as “0” months. Two samples designated “+3” and “+15” were drawn 3 and 15 months respectively following a diagnosis of a stage I head and neck cancer in one participant of the lung cancer screening study.

### Assay composition and procedures

Marker discovery, measurement and statistical analysis has been described previously [7]–[9]. The marker panel was comprised of six individual tumor-associated autoantibodies that offered robust discrimination between cancer and noncancer samples in prior analysis; these six also provided consistent performance as a combined measure in a single assay based on receiver operating characteristic area under the curve. T7-phage-expressed capture proteins were derived from cDNA tumor libraries [7]–[9]. These putative autoantibody markers corresponded to apurinic/apyrimidinic endonuclease-1 (APEX1), nucleolar and coiled-body phosphoprotein 1 (NOLC1), splicing factor 3a (SF3A3), paxillin (PXN), BAC clone R-580E16 (unknown protein product) and mitochondrial 16S ribosomal RNA (MT-RNR2). [7,8 and unpublished] All phage-expressed capture proteins were covalently bound to Luminex microspheres for multiplex analysis using commercially available protocols. Autoantibody levels were quantified using biotinylated anti–human IgG and R-phycoerythrin–labeled streptavidin. The mean absolute fluorescence to each marker was calculated from triplicate measurements for each sample. No-sample controls included in each run consistently measured near zero.

A single absolute fluorescence value was generated for each sample using the sum from individual markers. A cutoff value of 640, corresponding to the lower quartile (set specificity at 25%), would be expected to maximize capacity for detecting cancer at the earliest stages of disease while still providing an improved the ratio of scans performed to cancers detected. That cutoff was applied to class prediction in the Kentucky CT screening cohort. Relevant points of data analysis included distribution in the at risk population and comparability to the Mayo Clinic cohort, consistency of annual measures from individual subjects, accurate classification of cancer samples at the time of and prior to radiographic detection.

## Results

The additive sum of absolute fluorescence from six markers was used as an intuitive measure of overall autoantibody reactivity to provide a single value point for each sample, define distribution in the at risk population, and assign cutoffs for cancer prediction in an independent cohort. The median value across 180 non-cancer samples from the Mayo Clinic sample cohort was 1126 fluorescent units (FU), with 25%/75% quartile values of 640 and 2076 FU respectively; there was one extreme outlier. A cutoff of 640 fluorescent units offered 88% sensitivity across fifty cancer samples in the Mayo cohort, which included accurate classification of 6/6 established stage I cancers and 38/44 samples drawn one to five years prior to radiographic appearance. By comparison the median value across 237 non-cancer samples from the Kentucky cohort was 726 fluorescent units (FU), with 25%/75% quartile values of 461 and 1249 FU respectively, which is roughly one third lower than measured in the Mayo Clinic sample cohort. A contingency chart (table 2) shows class prediction in the Kentucky cohort at the predetermined cutoff of 640 FU, and also bares the effect of inflated cutoff values on sensitivity and specificity that resulted from the discrepancy between the training and testing cohorts. The cutoff of 640 FU accurately classified 102/237 non-lung cancer samples (43%) and 11/19 cancer samples (58%), which included 4/5 stage I lung cancers (80%), and 7/14 of occult cancer samples (50%) one to five years prior to radiographic appearance. Class prediction and temporal relationship of sample draw to cancer diagnosis is summarized in table 1.

**Table 2:** Contingency chart: class predictions by sample at various marker levels in the Kentucky screening cohort.

Diagnosis	No lung cancer		Screening and clinically diagnosed lung cancers			
Cutoff	Specificity		Sensitivity			
Absolute fluorescence	By sample (n = 237)	By case (n = 86)	By sample All cases: (n = 19)	By sample Stage I: (n = 5)	By sample Occult: (n = 14)	By case (n = 9)
500	31%	22%	58%	80%	50%	56%
600	41%	23%	58%	80%	50%	56%
**640**	**43%**	**28%**	**58%**	**80%**	**50%**	**56%**
700	48%	38%	53%	80%	50%	56%
800	54%	38%	53%	60%	43%	44%
900	60%	41%	53%	60%	43%	44%
1000	65%	52%	42%	60%	43%	44%
1500	81%	72%	37%	40%	43%	33%
2000	88%	82%	21%	40%	14%	22%
2500	93%	89%	21%	40%	14%	22%
3000	96%	92%	16%	40%	7%	22%
3500	97%	92%	16%	40%	7%	22%
4000	98%	97%	11%	20%	7%	11%

Specificity is presented by case series (all negative measures) and by individual sample (time of negative radiograph). Bolded data are predictions using predetermined cutoff value (640 FU). Absolute fluorescence is the additive sum of six markers in the panel.

Squamous and adenocarcinoma histologies were both represented among the true positives; there was nothing uniquely apparent about false negative samples. Other cancers accounted for 13/135 false positive measures (Table 1). Six of the seven independently diagnosed non-thoracic malignancies in the KY cohort measured positively in one or more annual samples. The single highest value was a subject lost to follow-up after prevalence screening who was diagnosed with extranodal marginal zone B-cell lymphoma (MALT) five years after enrollment. Benign intrathoracic findings were common to subjects with false positive and true negative measures. The majority of false positives represented persistent elevations across serial screening cycles. Among the 130 false positive samples (>640 FU) in subjects with at least two annual samples, only six (4.6%) were singular events within the series of two or more annual measures.

## Discussion

Primary objectives were to confirm the principles and precepts of autoantibody profiling and assess the potential of an autoantibody profile to increase efficiency and diagnostic accuracy of screening CT. Samples from the Mayo Clinic CT screening trial were used to define range and distribution of a composite measure within a screening population, and assign a cutoff value that would allow maximum sensitivity for lung cancers at and below the detectable limits of CT scanning. Distribution measures and relative cutoffs for cancer detection were tested in an independent screening cohort from the 5^th^ district of Kentucky. A cutoff set on the lower quartile of 180 noncancer controls in the Mayo cohort provided reliable detection of established stage I cancers and capacity to detect a percentage of incidence cancers prior to radiographic appearance in both cohorts. Observed frequency of serially positive and serially negative values across annual repeats in the Kentucky screening cohort suggests that autoantibody levels have a specific biologic basis even when there is no clinically apparent significance to the measure. The assay does not appear specific for lung cancer, although the variety of non-thoracic malignancies precludes any conclusion about histologic specificity.

Inflated cutoff values that resulted from the notable discrepancy in the quartile distributions between the two cohorts skewed the dynamic range towards specificity in the Kentucky cohort. Although demographics, differences in eligibility criteria of the two studies and numerous independent clinical variables could account for this discrepancy, neither cohort is adequately sized for multivariable stratification. Conversely, observed differences in two independent but uniformly collected, moderately large and relatively comparable sample sets point strongly to sample age, processing, handling and/or storage as a source of preclinical error. Specifically, distribution analysis and assignment of cutoff values based on archived samples from two high-risk cohorts seems likely to have identified a biological effect that might not have been recognized with alternate study designs. Despite the presumption that autoantibodies are resilient biomarkers, there is a paucity of data on the consistency of autoantibody measures under various storage conditions and durations. Albeit limited, literature indicates serum antibody levels increase in cryopreserved samples over years of storage, possibly related to antigen-antibody complex dissociation and protein degradation [19], [20]. Importantly, the current data shows how the validation process can be encumbered by variables unique to archived sample sets, which must be considered when transitioning from laboratory-based analysis to implementation in population-based applications.

Even when given allowance for quantifiable preclinical error and the effect of inflated cutoff values on predictive accuracy in the validation set, the data discourage more advanced validation. The appeal of detecting occult disease with lead-time advantage over CT scanning is tempered by excessive false negative rates, and certainly restricts this assay’s utility in selecting individuals that most warrant serial imaging [5]. Interpretation of positive measures is further confounded by the apparent lack of specificity for thoracic malignancy.

Provisional assessment of the small number of radiographically detectable cancers in post hoc analysis approximates that of autoantibody profiles independently validated by other groups testing for established cancers [21]–[23]. If by extension we assume the best achievable sensitivity for stage I cancer is 80%, with a corresponding specificity of 40% expanding our analysis to sample sets with larger number of established cancers does not seem warranted. Also similar to other assays in the literature, a provisional sensitivity of 40% for established disease corresponds to specificity >90% [21]–[24]. Adjusting the cutoff for high specificity seems only to further deviate from a conventional screening paradigm. If used to further stratify cases by probability of cancer, however, a cutoff that favors specificity could mitigate inter-reader variability and reduce the number of false negative readings on screening CT scans [25], [26]. A highly specific assay might also help discriminate benign from malignant nodules identified during screening, even though predictive value will be compromised by the promiscuity of the assay for both occult and radiographically apparent disease [27]. In summary, this report does not draw conclusions about future utility of this approach, but this validation study does not seem to support use of this assay as a primary population-based screening tool. Combining additional investigation with knowledge of this assay’s performance may identify other logical areas for autoantibody profiling in lung cancer diagnosis and management.

## References

[1] National Lung Screening Trial Research Team, Aberle DR, Adams AM, Berg CD, Black WC, et al (2011) Reduced lung-cancer mortality with low-dose computed tomographic screening. N Engl J Med 365: 395–409. 21714641 10.1056/NEJMoa1102873 PMC4356534

[2] Marshall E. Cancer screening (2010) The promise and pitfalls of a cancer breakthrough. Science 330: 900–1. 10.1126/science.330.6006.900-b 21071639

[3] Van't Westeinde SC, van Klaveren RJ (2011) Screening and early detection of lung cancer. Cancer J. 17: 3–10. 10.1097/PPO.0b013e3182099319 21263260

[4] Hanash SM, Baik CS, Kallioniemi O (2011) Emerging molecular biomarkers—blood-based strategies to detect and monitor cancer. Nat Rev Clin Oncol 8: 142–50. 21364687 10.1038/nrclinonc.2010.220

[5] Desmetz C, Mange A, Maudelonde T, Solassol J (2011) Autoantibody signatures: progress and perspectives for early cancer detection J Cell Mol Med. 15: 2013–2024. 10.1111/j.1582-4934.2011.01355.x PMC4394213 21651719

[6] Chapman CJ, Murray A, McElveen JE, Sahin U, Luxemburger U, et al (2008) Autoantibodies in lung cancer: possibilities for early detection and subsequent cure. Thorax 63: 228–33. 17932110 10.1136/thx.2007.083592

[7] Zhong L, Hidalgo GE, Stromberg AJ, Khattar NH, Jett JR, et al (2005) Using protein microarray as a diagnostic assay for non-small cell lung cancer. Am J Respir Crit Care Med 172: 1308–14. 16109979 10.1164/rccm.200505-830OC PMC2718416

[8] Zhong L, Coe SP, Stromberg AJ, Khattar NH, Jett JR, et al (2006) Profiling tumor-associated antibodies for early detection of non-small cell lung cancer. J Thorac Oncol 1: 513–9. 17409910

[9] Khattar NH, Coe-Atkinson SP, Stromberg AJ, Jett JR, Hirschowitz EA (2010) Lung cancer-associated auto-antibodies measured using seven amino acid peptides in a diagnostic blood test for lung cancer. Cancer Biol Ther 10: 267–72. 20543565 10.4161/cbt.10.3.12395 PMC3040837

[10] Backes C, Ludwig N, Leidinger P, Harz C, Hoffmann J, et al (2011) Immunogenicity of autoantigens. BMC Genomics 12: 340. 21726451 10.1186/1471-2164-12-340 PMC3149588

[11] Qiu J, Choi G, Li L, Wang H, Pitteri SJ, et al (2008) Occurrence of autoantibodies to annexin I, 14-3-3 theta and LAMR1 in prediagnostic lung cancer sera. J Clin Oncol 26: 5060–6. 18794547 10.1200/JCO.2008.16.2388 PMC2652098

[12] Lam S, Boyle P, Healey GF, Maddison P, Peek L, et al (2011) EarlyCDT-Lung: an immunobiomarker test as an aid to early detection of lung cancer. Cancer Prev Res 4: 1126–34. 10.1158/1940-6207.CAPR-10-0328 21733826

[13] Doyle HA, Mamula MJ (2001) Post-translational protein modifications in antigen recognition and autoimmunity. Trends Immunol 22: 443–9. 11473834 10.1016/s1471-4906(01)01976-7

[14] Trivers GE, De Benedetti VM, Cawley HL, Caron G, Harrington AM, et al (1996) Anti-p53 antibodies in sera from patients with chronic obstructive pulmonary disease can predate a diagnosis of cancer. Clin Cancer Res 2: 1767–1775. 9816128

[15] Chapman CJ, Robertson JFR, Murray A, Titulaer M, Lang B, et al (2010) The Presence of Autoantibodies to Tumour-Associated Antigens can Predate Clinical Diagnosis of Lung Cancer. Chest 138: s775A.

[16] Swensen SJ, Jett JR, Hartman TE, Midthun DE, Sloan JA, et al (2003) Lung cancer screening with CT: Mayo Clinic experience. Radiology. 226: 756–61. 10.1148/radiol.2263020036 12601181

[17] Swensen SJ, Jett JR, Hartman TE, Midthun DE, Mandrekar SJ, et al (2005) CT screening for lung cancer: five-year prospective experience. Radiology. 235: 259–65. 10.1148/radiol.2351041662 15695622

[18] Bensadoun E, Brooks M, Baron A, Mannino DM, Hirschowitz EA, et al. (2011) Marty Driesler Lung Cancer Project: Preliminary Report Of Lung Cancer Screening In Rural Kentucky. American Thoracic Society A17961.

[19] Männistö T, Surcel HM, Bloigu A, Ruokonen A, Hartikainen AL, et al (2007) The effect of freezing, thawing, and short- and long-term storage on serum thyrotropin, thyroid hormones, and thyroid autoantibodies: implications for analyzing samples stored in serum banks. Clin Chem 53: 1986–7. 17954505 10.1373/clinchem.2007.091371

[20] Kugler KG, Hackl WO, Mueller LA, Fiegl H, Graber A, et al (2011) The Impact of Sample Storage Time on Estimates of Association in Biomarker Discovery Studies. J Clin Bioinforma 1: 9. 21743835 10.1186/2043-9113-1-9 PMC3131186

[21] Boyle P, Chapman CJ, Holdenrieder S, Murray A, Robertson C, et al (2011) Clinical validation of an autoantibody test for lung cancer. Ann Oncol 22: 383–389. 20675559 10.1093/annonc/mdq361 PMC3030465

[22] Chapman CJ, Thorpe AJ, Murray A, Parsy-Kowalska CB, Allen J, et al (2011) Immunobiomarkers in small cell lung cancer: potential early cancer signals Clin Cancer Res. 17: 1474–1480. 10.1158/1078-0432.CCR-10-1363 21138858

[23] Chapman CJ, Healey GF, Murray A, Boyle P, Robertson C, et al (2012) EarlyCDT®-Lung Test: Improved Clinical Utility Through Additional Autoantibody Assays. Tumour Biol 33: 1319–1326. 22492236 10.1007/s13277-012-0379-2 PMC3460172

[24] Macdonald IK, Murray A, Healey GF, Parsy-Kowalska CB, Allen J, et al (2012) Application of a High Throughput Method of Biomarker Discovery to Improvement of the EarlyCDT(®)-Lung Test. PLoS One. 7: e51002. 10.1371/journal.pone.0051002 PMC3521770 23272083

[25] Singh S, Pinsky P, Fineberg NS, Gierada DS, Garg K, et al (2011) Evaluation of reader variability in the interpretation of follow-up CT scans at lung cancer screening. Radiology 259: 263–70. 21248232 10.1148/radiol.10101254 PMC3064819

[26] Gierada DS, Pilgram TK, Ford M, Fagerstrom RM, Church TR, et al (2008) Lung cancer: Interobserver agreement on interprestion of pulmonary findings at low-dose CR screening. Radiology 246: 265–272. 18024436 10.1148/radiol.2461062097

[27] Rom WN, Goldberg JD, Addrizzo-Harris D, Watson HN, Khilkin M, et al (2010) Identification of an autoantibody panel to separate lung cancer from smokers and nonsmokers. BMC Cancer 10: 234. 20504322 10.1186/1471-2407-10-234 PMC2885364

